# Immunological consequences of arsenic trioxide-induced necrosis

**DOI:** 10.1038/s41423-023-00976-4

**Published:** 2023-01-25

**Authors:** Shubhangi Gavali, Wulf Tonnus, Andreas Linkermann

**Affiliations:** 1 Division of Nephrology, Department of Internal Medicine 3, University Hospital Carl Gustav Carus at the Technische Universität Dresden, Dresden, Germany; 2 Division of Nephrology, Department of Medicine, Albert Einstein College of Medicine, Bronx, NY USA

**Keywords:** Cancer, Cell death and immune response

**Fig. 1:**
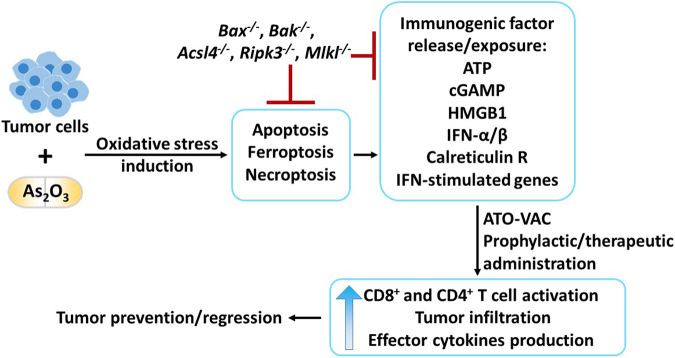
Arsenic trioxide (As_2_O_3_, ATO)-induced immunogenic cell death in tumors as a tool to elicit antitumor immunity. Whole-cell tumor vaccines generated using cytotoxic drugs ex vivo offer an attractive approach to target virus-irrelevant neoplasms. Applying this knowledge, Chen et al. explored the prophylactic and therapeutic efficacy of an ATO-based whole-cell vaccine. Preconditioning of tumor cells with ATO induces oxidative stress, which, simultaneously or sequentially, triggers cell death pathways such as apoptosis, ferroptosis and necroptosis. These pathways are associated with the release or exposure of numerous immunogenic factors that activate the immune system. Deletion of executors of each of the involved death pathways inhibited tumor cell death and thus blocked the release of immunogenic factors. The prophylactic and therapeutic administration of an ATO-based whole-cell vaccine (ATO-VAC) successfully induced an adaptive immune response. Thus, a strong antitumor response in the recipient mice was elicited by creating a highly immunogenic tumor microenvironment that was flooded with activated tumor-infiltrating CD8^+^ and CD4^+^ T cells with heightened effector cytokine production, leading to tumor prevention or regression

The exact mechanism of arsenic trioxide (ATO, As_2_O_3_) poisoning has been a mystery for centuries. While rumors even suggest that it might have killed Napoleon, more recent work suggests ATO as a potential treatment option for acute promyelocytic leukemia [1]. While the potency of ATO in cell killing has been recognized, hardly anyone has considered the immunological consequences of cell death in tumors or tissues following ATO treatment. In this issue of *Cellular and Molecular Immunology*, Chen et al. address this question, adding yet another chapter to the complex relation between cell death and the immune response (Fig. 1) [2].

In their current work, Chen et al. started their investigation by setting up multiparameter drug screening for lethal antineoplastic drugs, including ATO [2]. Evaluation of the effect on the viability of TC-1 lung cancer cells showed that ATO exhibited potent cytotoxicity in a dose- and time-dependent manner. Preconditioning of OVA-expressing TC-1 cells with ATO before subcutaneous injection into naive C57Bl/6 mice increased tumor immunogenicity, as evidenced by accelerated OT-1 cell proliferation. Furthermore, coculture of ATO-preconditioned TC-1 OVA cells with bone marrow-derived dendritic cells and OT-1 cells, mimicking tumor antigen presentation ex vivo, resulted in a surge in IFN-γ secretion by OT-1 cells. ATO displayed cytotoxic effects against various solid and hematopoietic tumor cell lines, suggesting ATO-mediated killing as a potent immunogenic cell death (ICD) inducer and ATO as a broad-spectrum cytotoxic agent. The authors proceeded to examine the prophylactic efficacy of an ATO-based whole-cell vaccine (ATO-VAC). ATO-VAC effectively protected mice from rechallenge with live TC-1 cells. This prophylactic effect appeared to be mediated by CD8^+^ T cells. To elucidate the mechanism of antitumor activity induced by ATO-VAC, the authors analyzed the pattern of ICD factors accompanying ATO-induced cell death. ATO treatment indeed created an exceedingly immunogenic environment through the exposure/release of a myriad of ICD factors. The effects included the release of ATP, cGAMP, and HMGB1 into the extracellular space; translocation of calreticulin to the plasma membrane; enhanced production of IFN-α/β; and transcription of interferon-stimulated genes. Transcriptomic profiling suggested the induction of oxidative stress in ATO-treated tumor cells, which was blocked by a ROS scavenger. To delineate the key pathways involved, western blot analysis of proteins central to autophagy, apoptosis, pyroptosis, ferroptosis, and necroptosis in TC-1 cells was performed. Interestingly, ATO simultaneously or sequentially activated autophagy, apoptosis, ferroptosis, and necroptosis with the possibility of crosstalk among these pathways. ATO-triggered oxidative stress proved indispensable for activating these pathways since the ROS scavenger NAC inhibited ATO-induced cell death, suggesting that oxidative triggers function upstream in this sequence. KO clones lacking each cell death executor were created using CRISPR‒Cas9 technology. Mice receiving *Acsl4*^*-/-*^, *Ripk3*^*-/-*^, or *Mlkl*^*-/-*^ whole-cell vaccines displayed a low frequency of adoptively transferred OT-1 cells in the popliteal lymph nodes. The KO clones for apoptotic, necroptotic and ferroptotic factors showed impaired ATO-induced extracellular ATP accumulation and exposure of calreticulin on the plasma membrane. In addition, *Acsl4*-dependent ferroptosis and *Ripk3-* and *Mlkl*-dependent necroptosis seemed to contribute to cGAMP generation and release, suggesting pivotal roles in ATO-induced ICD and the prophylactic efficacy of the vaccine. Notably, ATO-VAC showed substantial therapeutic potential, as the administration of this vaccine to TC-1 cell-bearing mice significantly reduced tumor growth. As expected, ferroptotic and necroptotic pathways played key roles, as evidenced by reduced levels of various immune cells in the tumor microenvironment (TME) and reduced expression of an early activation marker (CD69) and effector cytokines (IFN-γ and TNF-α) by tumor-infiltrating CD8^+^ and CD4^+^ T cells in *Acsl4*^*-/-*^, *Ripk3*^*-/-*^ and *Mlkl*^*-/-*^ mice. ATO-VAC provides a promising agent for creating an immensely immunogenic TME and produces an even stronger antineoplastic effect when combined with immune checkpoint inhibitors, such as anti-PD-1.

These results suggest the involvement of various pathways of regulated necrosis. However, a more detailed understanding of the sequential pathway stimulation and the precise triggers and, most importantly, performance of the missing safety assessment for ATO-VAC preclude advancing this approach closer to a clinical test. In addition, not all necrotic-type cell death pathways are immunogenic by nature. Indeed, recent data suggest that ferroptotic cell death can inhibit the antitumor response [3] and prevent cross-presentation by conventional type 1 dendritic cells [4]. In contrast, necroptosis promotes cross-presentation [5]. Therefore, understanding the individual components and exactly how they shape the immune response of each cell death pathway is mandatory to further understand phenomena like the one reported here [2]. The relevance of this understanding goes far beyond the field of oncology, as similar generation of necrotic debris has been reported to occur during myocardial infarction [6], acute kidney injury [7] and solid organ transplantation [8]. One possible way to untangle the individual cell death pathways is to learn from studies on drugs, such as the immunosuppressant dexamethasone, and investigate its direct effects on cell death [9]. Future understanding of the basic signaling within these pathways may pave the way to future treatments harnessing knowledge about the immunogenicity of regulated necrosis.
